# The Flavones Apigenin and Luteolin Induce FOXO1 Translocation but Inhibit Gluconeogenic and Lipogenic Gene Expression in Human Cells

**DOI:** 10.1371/journal.pone.0104321

**Published:** 2014-08-19

**Authors:** Christiane Bumke-Vogt, Martin A. Osterhoff, Andrea Borchert, Valentina Guzman-Perez, Zeinab Sarem, Andreas L. Birkenfeld, Volker Bähr, Andreas F. H. Pfeiffer

**Affiliations:** 1 Department of Clinical Nutrition, German Institute of Human Nutrition, Potsdam-Rehbrücke, Nuthetal, Germany; 2 Department of Endocrinology, Diabetes and Nutrition, Charité - University Medicine Berlin, Berlin, Germany; 3 Department of Nutrition and Biochemistry, Sciences Faculty - Pontificia Universidad Javeriana, Bogota D.C., Colombia; GDC, Germany

## Abstract

The flavones apigenin (4′,5,7,-trihydroxyflavone) and luteolin (3′,4′,5,7,-tetrahydroxyflavone) are plant secondary metabolites with antioxidant, antiinflammatory, and anticancer activities. We evaluated their impact on cell signaling pathways related to insulin-resistance and type 2 diabetes. Apigenin and luteolin were identified in our U-2 OS (human osteosarcoma) cell screening assay for micronutrients triggering rapid intracellular translocation of the forkhead box transcription factor O1 (FOXO1), an important mediator of insulin signal transduction. Insulin reversed the translocation of FOXO1 as shown by live cell imaging. The impact on the expression of target genes was evaluated in HepG2 (human hepatoma) cells. The mRNA-expression of the gluconeogenic enzymes phosphoenolpyruvate carboxykinase (PEPCK) and glucose-6-phosphatase (G6Pc), the lipogenic enzymes fatty-acid synthase (FASN) and acetyl-CoA-carboxylase (ACC) were down-regulated by both flavones with smaller effective dosages of apigenin than for luteolin. PKB/AKT-, PRAS40-, p70S6K-, and S6-phosphorylation was reduced by apigenin and luteolin but not that of the insulin-like growth factor receptor IGF-1R by apigenin indicating a direct inhibition of the PKB/AKT-signaling pathway distal to the IGF-1 receptor. N-acetyl-L-cysteine did not prevent FOXO1 nuclear translocation induced by apigenin and luteolin, suggesting that these flavones do not act via oxidative stress. The roles of FOXO1, FOXO3a, AKT, sirtuin1 (SIRT1), and nuclear factor (erythroid-derived2)-like2 (NRF2), investigated by siRNA knockdown, showed differential patterns of signal pathways involved and a role of NRF2 in the inhibition of gluconeogenic enzyme expression. We conclude that these flavones show an antidiabetic potential due to reduction of gluconeogenic and lipogenic capacity despite inhibition of the PKB/AKT pathway which justifies detailed investigation *in vivo*.

## Introduction

The prevalence of type 2 diabetes (T2DM) and associated co-morbidities is rising worldwide [1]. Obesity is a main risk factor [2] and a feature of the metabolic syndrome that includes hypertension, dyslipidemia, glucose intolerance and insulin resistance [3]. The prevention of type 2 diabetes by life style intervention including diet and exercise was successful [4]. To clarify the preventive impact of macronutrient compositions, the effect of supplemented isoenergetic diets differing in cereal fiber and protein content on insulin sensitivity was investigated in overweight humans [5]. Consumption of higher quantities of vegetable and a variety of fruits and vegetables containing bioactive phytochemicals were associated with a reduced risk of T2D in a case cohort study of EPIC-Norfolk [6]. Micronutrients such as the polyphenol resveratrol (3,5,4′-trihydroxystilbene) showed controversial outcomes in different studies from improvement of insulin sensitivity [7], [8], [9] to absence of any evidence of metabolic effects [10]. Mediterranean diets rich in polyphenols were shown to prevent T2DM [11].

To screen plant derived micronutrients for their potential antidiabetic activity we analyzed their impact on the intracellular distribution of FOXO1 indicating the state of insulin signaling. The forkhead box transcription factor FOXO1 undergoes nuclear exclusion induced by insulin via the phosphatidylinositol 3′-kinase (PI3K)-PKB/AKT pathway which leads to an inactivating phosphorylation of FOXO1 at Ser256 (gatekeeper), Thr24, and Ser319 reducing tight DNA-binding and enhancing the binding of the protein 14-3-3 followed by nuclear export and cytoplasmic retention of FOXO1 [12]. FOXO activity and intracellular localization is also controlled by the acetylation/deacetylation state [13], [14], [15]. Deacetylation of FOXO by the silent information regulator 2 (Sir2) mammalian ortholog sirtuin 1 (SIRT1) makes FOXO available for activating monoubiquitinylation [16] and inactivating polyubiquitinylation by E3 ubiquitin-protein ligase MDM2 inducing FOXO intranuclear and nuclear-cytoplasmic shuttling respectively [17]. These multiple upstream pathways regulating FOXO activity through posttranslational modifications (PTMs) and nuclear-cytoplasmic shuttling enable FOXOs to act as signaling integrators for homeostasis maintenance, orchestrating metabolic target genes for gluconeogenesis, food intake and redox balance, protein homeostasis including autophagy, cell cycle arrest, and apoptosis in response to metabolic stress, oxidative stress and growth factor deprivation by differential downstream transactivations [18]. Localization of the forkhead box transcription factor FOXO in the nucleus is itself indicative of, but not identical to, transcriptional activity, as several FOXO regulators also display dynamic localization [18]. Using a FOXO1-translocation assay, we found highest translocation activity induced by apigenin (4′,5,7,-trihydroxyflavone) and luteolin (3′,4′,5,7,-tetrahydroxyflavone) in our screening of a library of plant micronutrients. Luteolin is one of the most common polyphenolic flavonoids present in many edible plants such as carrots, peppers, celery, olive oil, peppermint, thyme, rosemary, oregano, lettuce, pomegranate, artichoke, cucumber, lemon, beets, cabbage, cauliflower, spinach, parsley, and green tea [19]. A high dietary intake of luteolin was associated with a lowered risk of acute myocardial infarction [20]. Many luteolin containing plants possess antioxidant properties associated with the capacity to scavenge reactive oxygen and nitrogen species (ROS, RNS) to inhibit pro-oxidant enzymes and to induce antioxidant enzymes observed *in vitro* and *in vivo* by this flavonoid as reviewed by Lopez-Lazaro [19]. Regarding anti-inflammatory activities, luteolin was identified as the most potent flavonoid in inhibiting TNF-alpha release from macrophages, in blocking lipopolysaccharide (LPS)-induced activation of the nuclear factor-kappa B (NF-kappa B) and IL-6 production by inhibition of the JNK and AP1-signaling pathways [21]. Apigenin with three phenolic hydroxyl groups has a lower TEAC (Trolox equivalent antioxidant capacity) than luteolin bearing four OH-groups including an ortho-dihydroxy structure in the B ring which is essential as well as the 2,3-doublebond in conjugation with the 4-oxo function in the C ring and the 5- and 7-OH groups in ring A for effective free radical scavenging by dissociation of hydroxyl functions [22]. Like luteolin, apigenin is a natural plant flavone abundantly common in chamomile, parsley, onions, grapefruit, oranges and plant derived beverages with antioxidative and antiproliferative effects in human cancers of the breast, cervix, colon, lung, ovary, prostate, skin, thyroid, and liver [23]. With FOXO1 not only as the endpoint of IGF signaling but also of insulin signaling via the PI3K-AKT pathway, we focused our investigations on the metabolic effects of apigenin and luteolin to analyze their roles in modulating insulin signal transduction which is disturbed in insulin resistance and T2D. To date, there has been no report on the regulation of gluconeogenesis and lipogenesis by flavones.

Health promoting effects on preclinical diabetes by reduction of blood glucose by attenuation of gluconeogenesis, which is elevated during insulin resistance, have not been described for flavones to date. We found apigenin and luteolin to induce FOXO1 translocation, which plays a key role in insulin signaling, using stably transfected human U-2 OS cells expressing FOXO1-GFP. For analysis of FOXO-target gene expression, we analyzed HepG2 cells expressing hepatic PEPCK and G6Pc and showed for the first time a down-regulation of mRNA of key gluconeogenic enzymes by apigenin and luteolin in a dose dependent manner thereby providing an antihyperglycemic effect. The gene expression of the lipogenic enzymes FASN and ACC was reduced by these flavones potentially preventing hepatic steatosis. To identify different signaling pathways involved in these effects, gene expression analyses were performed after knock down of the transcription factors NRF2, FOXO3a, FOXO1, the downstream deacetylase SIRT1 and the upstream modulating kinase AKT as well. Remarkably, apigenin and luteolin also reduced the insulin-induced phosphorylation of AKT, mammalian target of rapamycin (mTOR), p70S6K, the ribosomal protein S6, and the proline-rich AKT/PKB substrate 40 kDa (PRAS40) indicating an inhibition of the AKT signaling pathway.

## Material and Methods

### Chemicals

Apigenin (≥97% purity, from parsley), luteolin (≥98% purity), isokaempferide (≥90% purity), kaempferol (≥90% purity), quercetin (≥95% purity), and resveratrol (≥99% purity) were purchased from Sigma-Aldrich and solubilized in dimethyl sulfoxide (DMSO). In all experiments the final concentration of DMSO did not exceed 0.5%. Insulin (human recombinant) 10 mg/ml solution in 4-(2-hydroxyethyl)-1-piperazineethanesulfonic acid (HEPES) pH 8.2 and N-acetyl-L-cysteine (NAC) were obtained from Sigma-Aldrich.

### Preparation of solutions

Apigenin, luteolin, isokaempferide, kaempferol, quercetin, and resveratrol were dissolved in DMSO to obtain a 20 mM stock solution and diluted in medium for cell culture (DMEM for U-2 OS and EMEM for HepG2) to obtain 100 µM flavone as highest concentration for stimulation. Further dilutions down to 0.5 µM for dose-response experiments were performed with addition of DMSO to keep its concentration stable at 0.5%, which was used for mock stimulation in the control cells as well. Insulin was diluted from 10 mg/ml ( = 1,722 mM) in medium to obtain 100 nM final concentration for stimulation of cells in culture.

### Cell lines and culture

U-2 OS (human osteosarcoma cell line) was purchased from ECACC (European Animal and Cell Collection). Cells were maintained using DMEM (Dulbecco's Modified Eagle Medium) with 4.5 g/L D-glucose, 3.7 g/L NaHCO_3_, stable glutamine, and Na-pyruvate supplemented with 10% FBS (Fetal Bovine Serum) and kept viable in a humidified atmosphere, at 37°C, 5% CO_2_. HepG2 (hepatocellular carcinoma, human) was purchased from ECACC and cultivated in EMEM (EBSS Eagle's Minimum Essential Medium with Earle's Balanced Salt Solution), stable glutamine, 2.2 g NaHCO3, 1 g/L D-glucose, supplemented with 1% NEAA (Non-Essential Aminoacids) and 10% FBS purchased from Biochrom Germany. A human embryonic kidney (HEK) cell line stably transfected with the human IGF-1R was a gift from Prof. J. Frystyk, institute of Clinical Medicine, Aarhus University, Denmark. Cells were maintained in DMEM (Biochrom AG, Berlin, Germany) supplemented with 10% fetal bovine serum, 1% penicillin/streptomycin, 1% hygromycin and 0.1% geneticin. For passaging of cells TrypLE express from life Technologies was used.

### Cell treatment with apigenin, luteolin, isokaempferide, kaempferol, resveratrol, and insulin

Before incubation of cells with test compounds, growth of sub-confluent U-2 OS cells was calmed down for 16 h by reduction of 10% FBS to 2% FBS and further to 0% FBS in DMEM for 1 h starvation. HepG2 cells were starved for 16 h in EMEM with 1 g/L glucose without FBS. Stimulation of cells was performed in FBS-free medium with apigenin, luteolin, isokaempferide, kaempferol, and resveratrol dissolved in DMSO and diluted in medium (final DMSO concentration ≤0.5%). Control cells were incubated with the same amount of DMSO (mock stimulation). Insulin was applied to DMSO pretreated and untreated cells.

### Cell transfection

Transfection of sub-confluent cells with DNA plasmids was performed with Lipofectamine 2000 in Opti-MEM reduced serum medium from Invitrogen according to the manufacturer's description. The vector for stable transfection of U-2 OS for constitutive expression of GFP- labeled FOXO1 was pEGFP-N1 with an insertion of FOXO1 cDNA. Zhang et al. [24] subcloned the wild type sequence reported in GenBank AF032885 for full length human FKHR = FOXO1 complete coding sequence (CDS) + 224 base pairs (bp) of the 5′- untranslated region (UTR) and 133 bp of 3′-UTR into the XbaI-AccI site in pAlter.MAX from Promega for site-directed mutagenesis converting sequences coding for serine-256, serine-319 and threonine 24 to alanine coding sequences. This was cloned in frame with the CDS of GFP into the BGLII-SalI site in pEGFP-N1 from Clontech for studies of cellular trafficking of mutated forms of FKHR. These constructs encoding mutant FKHR with the stop codon removed, followed by the C-terminal green fluorescent protein (GFP) tag (pEGFP-FOXO1mut) and pAlter.MAX -FOXO1wt (wild type) were kindly provided by Terry Unterman (University of Illinois at Chicago, USA). We excised the region with 3 mutations of FOXO1 from pEGFP-FOXO1mut by restriction with HindIII and BstEI and inserted the HindIII-BstEI-fragment without mutations from pAlter.MAX-FOXO1wt. The resulting pEGFP-FOXO1 contained the complete FOXO1 CDS + 33 bp of the 5′-UTR and according to our sequencing data the last triplet of the CDS ggc coding for glycine in front of the stop-codon tga was exchanged to tcg coding for serine followed by 33 bp coding for 11 connecting amino acids in front of the CDS of GFP.

Following transfection of pEGFP-FOXO1, U-2 OS cells were grown in DMEM + 10% FBS and Geneticin G418 sulfate from Calbiochem (0.4 mg/ml) was added after 48 h for selection of genetically engineered cells carrying the plasmid with encoded resistance. Separating cells over 3 passages by dilution up to 1∶10^8^ in 96-well plates coated with Poly-D-lysine starting with growth in conditioned medium resulted in homogeneous cell colonies expressing FOXO1-GFP, analyzed by fluorescence microscopic live cell imaging used for selection of GFP-positive cells.

Transfection of silencing RNA (siRNA) was performed using DharmaFECT4 (D4) transfection reagent proposed for HepG2 transfection by Dharmacon Thermo scientific with ON-TARGETplus SMARTpool siRNA including four SMARTselection-designed siRNAs pooled for efficient knockdown of each target: FOXO1, FOXO3a, AKT, SIRT1, NRF2, and NT (siGENOME Non Targeting)-siRNA. Target specific siRNA sequences were:

5′-GCGCUUAGACUGUGACAUG-3′; 5′-GAGGUAUGAGUCAGUAUAA-3′;

5′-UGACUUGGAUGGCAUGUUC-3′; 5′-GGACAACAACAGUAAAUUU-3′ for FOXO1;

5′-UAACUUUGAUUCCCUCAUC-3′; 5′-CGAAUCAGCUGACGACAGU-3′;

5′-GUACUCAACUAGUGCAAAC-3′; 5′-GCACAGAGUUGGAUGAAGU-3′ for FOXO3a;

5′-CAUCACACCACCUGACCAA-3′; 5′-ACAAGGACGGGCACAUUAA-3′;

5′-CAAGGGCACUUUCGGCAAG-3′; 5′-UCACAGCCCUGAAGUACUC-3′ for AKT;

5′-GCAAAGGAGCAGAUUAGUA-3′; 5′-GCGAUUGGGUACCGA-3′;

5′-GGAUAGGUCCAUAUACUUU-3′; 5′-CCACCUGAGUUGGAUGAUA-3′ for SIRT1;

5′-CACCUUAUAUGUCGAAGUU-3′; 5′-UGGAGUAAGUCGAGAAGUA-3′;

5′-GAGUUACAGUGUCUUAAUA-3′; 5′-UAAAGUGGCUGCUCAGAAU-3′ for NRF2

and Non-Targeting Sequences NT were:

#2 5′-UAAGGCUAUGAAGAGAUAC-3′; #3 5′-AUGUAUUGGCCUGUAUUAG-3′;

#4 5′-AUGAACGUGAAUUGCUCAA-3′; #5 5′-UGGUUUACAUGUCGACUAA-3′. Transfections of HepG2 were performed 24 h after seeding 100,000 cells per well of 12 well plates in EMEM with FBS 10%. SiRNAs were dissolved and diluted according to the manufacturer's description. Transfection reagent D4 was diluted in EMEM without FBS, incubated 5′ at RT and mixed with siRNAs followed by 20′ incubation at RT. HepG2 cells obtained fresh EMEM with 10% FBS before transfection. Cells were treated with transfection mixtures of D4 and NT-siRNA for transfection controls, or D4 and smart pool siRNAs for FOXO1, FOXO3a, AKT, SIRT1, NRF2 or mixtures of FOXO1/FOXO3a, FOXO1/NRF2, FOXO1/AKT, FOXO1/SIRT1, FOXO3a/SIRT1 and AKT/NRF2 for specific target knockdowns in triplicates. Because 24–48 h was recommended for RNA- knockdown and 48–96 h for protein-knockdown, we exchanged the medium to EMEM without FBS 32 h after transfection followed by 16 h starvation before treatment of the transfected cells with DMSO 0.1%, apigenin 20 µM and luteolin 20 µM for 24 h.

### RNA isolation and real-time PCR

Extraction of RNA was performed with the Nucleospin RNA II total RNA isolation kit from Machery-Nagel following treatment of cells with compounds as per figure legends. In each 1 µg of RNA was reverse transcribed with the High Capacity cDNA Reverse Transcription Kit from Applied Biosystems and quantitative real time PCR (qRT-PCR) was performed with the Power SYBR green PCR Master Mix from Applied Biosystems using the Vii7 Real-Time PCR System from Life Technologies. Each reaction was carried out in triplicates, using a standard curve with the relevant cDNA for each primer pair. Primers for qRT-PCR were designed using Primer Express and purchased from Invitrogen. Primer sequences used for real time PCR are summarized in Table 1. Amplification of 60S ribosomal protein L32 (RPL-32) housekeeping gene was performed for normalization of target gene expression.

**Table 1:** Primer sequences for qRT-PCR.

Primers for qRT-PCR targets	Oligo forward	Oligo sequence (5′ to 3′)	Oligo reverse	Oligo sequence (5′ to 3′)
60S ribosomal protein L32	hRLP-32_fwd	CAACGTCAAGGAGCTGGAAGT	hRLP-32_rev	TTGTGAGCGATCTCGGCAC
Acetyl-CoA-carboxylase	hACC_fwd	TCGCTTTGGGGGAAATAAAGTG	hACC_rev	ACCACCTACGGATAGACCGC
Fattyacid-synthetase	hFASN_fwd	AGACACTCGTGGGCTACAGCAT	hFASN_rev	ATGGCCTGGTAGGCGTTCT
Forkhead box O1	hFOXO1_fwd	GGCTGGAAGAATTCAATTCGTC	hFOXO1_rev	ACCCTCTGGATTGAGCATCCAC
Forkhead box O3	hFOXO3_fwd	CATGGCAAGCACAGAGTTGGA	hFOXO3_rev	CGGCTTGCTTACTGAAGGTGAC
Glucose-6-phosphatase	hG6Pc_fwd	CCCCTGATAAAGCAGTTCCCT	hG6Pc_rev	ATACACCTGCTGTGCCCATG
Nuclear factor (erythroid derived)-like2	hNRF2_fwd	AACTACTCCCAGGTTGCCCA	hNRF2_rev	CAAGTGACTGAAACGTAGCCGA
Phosphoenolpyruvat-carboxykinase	hPEPCK_fwd	AAGTATGACAACTGCTGGTTGGC	hPEPCK_rev	ATAACCGTCTTGCTTTCGATCCT
Proteinkinase B/AKT	hAKT1_fwd	GCTTCTATGGCGCTGAGATTGT	hAKT1_rev	TGATCTTAATGTGCCCGTCCTT
Sirtuin1	hSIRT1_fwd	ATGCTGGCCTAATAGAGTGGCA	hSIRT1_rev	CCTCAGCGCCATGGAAAAT

Primer design by Primer Express for qRT-PCR based on human mRNA sequences.

### Path Scan intracellular signaling array

We used the PathScan Intracellular Signaling Array Kit from Cell Signaling Technology with a fluorescent readout for simultaneous detection of 18 signaling molecules when phosphorylated such as AKT(Thr308), AKT(Ser473), AMPKα(Thr172), mTOR(Ser2448), p70S6K(Thr389), S6 Ribosomal Protein(Ser235/236), PRAS40(Thr246), GSK-3β(Ser9), BAD(Ser112), HSP27(Ser78), p53(Ser15), p38(Thr180/Tyr182), ERK1/2(Thr102/Tyr204), Stat1(Tyr701), Stat3(Tyr705) or cleaved PARP(Asp214) and Caspase-3(Asp175). Lysates were obtained from HepG2 cells treated with DMSO 0.1%, insulin 100 nM, insulin + DMSO, apigenin 20 µM, luteolin 20 µM, insulin + apigenin and insulin + luteolin for 30′, using the provided 1× cell lysis buffer supplemented with phenylmethylsulfonyl fluoride (PMSF) to a final concentration of 1 mM and phosphatase-inhibitor from Roche (1 tablet per 10 ml as recommended). Following ultrasonication and centrifugation the clear supernatant was used for the determination of protein contents with the BCA protein assay kit from BIO RAD. Samples were diluted in array diluent buffer to 1 mg/ml. Glass slides with antibody spotted nitrocellulose-pads were connected with a multi-well gasket for blocking each pad with 100 µl array blocking buffer per well for 15 minutes followed by 16 h incubation at 4°C with each 75 µl diluted lysate. After four washing steps with each 100 µl array wash buffer pads were incubated with 75 µl of the provided detection antibody cocktail for 1 h at RT, washed four times and incubated with 75 µl DyLight 680-linked streptavidin for 30 minutes under light protection. After four washing cycles each 5′ the gasket was removed, the slide rinsed in deionized water and dried completely. Slides were scanned with the LI-COR Bioluminescence imager and analyzed using ODYSSEY software.

### IGF-1 kinase receptor cell based activation assay

IGF-1 receptor phosphorylation was investigated as described [25] by a kinase receptor activation assay (KIRA) using a human embryonic kidney (HEK) cell line stably transfected with the human IGF-1R. Cells were maintained in DMEM supplemented with 10% fetal bovine serum, 1% penicillin/streptomycin, 1% hygromycin and 0.1% geneticin for 24 h. The medium was changed to DMEM with 0.1% human serum albumin (HSA) for further 24 h. After 48 h of cell culture maintenance, the cells were stimulated with different concentrations of human recombinant IGF-1 for 16 min at 37°C to obtain a standard curve. To clarify the effect of apigenin on IGF-1 receptor phosphorylation, the cells were stimulated with different concentrations of IGF-1 with or without apigenin 20 µM. Stimulated cells were lysed and transferred to a normal ELISA-sandwich assay using a mouse monoclonal IGF-1R antibody as a capture antibody and anti-mouse horseradish peroxidase-conjugated anti-phosphotyrosine monoclonal antibody as a detection antibody. All measurements were performed in duplicates.

### Cell viability assay

A CellTiter96 Aqueous Cell Proliferation assay from Promega was used to test the viability of HepG2 during 24 h incubation with apigenin and luteolin in the range of 1–100 µM including 0.5% of DMSO solvent, DMSO 0.5% alone and without DMSO. 20,000 HepG2 cells grown in MEM + 10% FBS were seeded per well in a clear 96-well plate without coating from Greiner. 5 h after seeding medium was exchanged using MEM without FBS for 16 h starvation. Apigenin and luteolin were added in triplicates per concentration 1 µM, 2 µM, 5 µM, 10 µM, 20 µM, 50 µM, 100 µM and cells incubated for 24 h. Following application of 20 µM (3-(4,5-dimethylthiazol-2-yl)-5-(3-carboxymethoxy phenyl)-2-(4-sulfophenyl)-2H-tetrazolium inner salt (MTS)/phenazine methosulfate (PMS - electron coupling reagent) per well, cells were incubated 4 h at 37°C under 0.5% CO_2_ and the optical density of the MTS bioreduction product formazan was measured after 4 h at 490 nm with the Wallac VICTOR plate reader.

### FOXO1 translocation assay

Transfected U-2 OS cells with pEGFP-FOXO1 stably expressing wild type FOXO1 tagged at the C-terminus with GFP were used for FOXO1-GFP visualization by fluorescence microscopy. 10,000–15,000 cells/100 µl DMEM + 10% FBS per well were seeded in black, clear bottom 96-well plates from BD coated with poly-D-lysine. After 6 h medium was exchanged to DMEM + 2% FBS and after 16 h to DMEM without FBS for 1 h of starvation before treatment of cells with test substances such as apigenin, luteolin, isokaempferide, kaempferol, quercetin, resveratrol, and other plant derived micronutrients solely and/or in combination with insulin in triplicates. Life cell imaging was performed with the Zeiss “Axio Observer.Z1” inverted microscope in a cell incubation chamber. Images with a filter for GFP were taken every minute up to 1 h to follow the intracellular translocation of FOXO1-GFP. For defining nuclear areas, cells were fixed with 4% paraformaldehyde in phosphate buffered saline (PBS) and nuclei stained with 4′,6-diamidino-2-paraphenylindole (DAPI) from Invitrogen 200 nM in 0.3% TritonX-100 for 30 min, washed in PBS, and exposed with the filter for DAPI as well. For quantification of GFP signals we used the BD Pathway 435 Bioimager from Becton Dickinson with a high performance laser-based autofocus for automated imaging of each of 96 wells with two exposures in the GFP-channel for separate detection in nuclear and cytoplasmic regions and one in the DAPI-channel. Using BD AttoVision version 1.6/435, images were acquired for GFP-Nuc and GFP-Cyto with exposures for 0.5 seconds each and for DAPI with 0.05 seconds per probe cycle for each well with a montage capture setup 2×2. For the multi-well plate setup a compound macro (nuclear translocation GFP GFP DAPI 96 well) was created resulting in a macro setup starting with a laser autofocus step followed by a cycle with three exposures: twice in the channel for GFP and once in the DAPI channel repeated for each well. A treatment plate map with definitions of substances and concentrations used for stimulations of cells per well in quadruplicates was set up to enable analysis of each experiment. Processing was performed under flat field correction and background subtraction. For the segmentation of cells we used the provided method “Cyto-Nuc Ring Band” with the shape “Ring (2outputs) Band” analyzing the DAPI channel for defining nuclear areas. In each exposure nuclei were split by watershed and an erosion factor of 7, scrapped to object pixels 400–2,000 and the inner nuclear region output reduced by an erosion width of 1 pixel. The cytoplasmic ring was obtained by a dilation of 4 pixels and an erosion of 3 pixels with an outer region output for a cytoplasmic area around the nucleus in a band excluding nuclear-cytoplasmic boundary from measurement of GFP intensities. Further analyses were performed with the BD Image Data Explorer from BD Biosciences using the measured regions of interest (Roi)Summary Re-analysis and treatment plate map as data sources to create the explorer data file. In the data management the treatment plate map was connected with the RoiSummary, wells for analysis selected, in parameter arithmetic a new parameter (GFP_Nuc_intensity/GFP_Cyt_intensity) as (Ratio Nuc/Cyt) added, and in constrains for cytoplasm GFP-intensities selected >800 above the background to exclude cells without FOXO-GFP expression and for nuclei <4,000 to exclude artifacts. Ratios Nuc/Cyt were evaluated in charting by bar charts with average parameter values per well, standard error over selected wells, and error bar charts by dose defined in treatment plate map. Ratios <1 represented cells with a predominant cytoplasmic localization of FOXO1-GFP and ratios >1 showed a nuclear accumulation of FOXO1. By normalization versus untreated cells, an induced translocation factor was calculated for treatment substances.

### Data handling and statistical analysis

All fluorescent microscopic data were analyzed using charts data from the BD image data explorer. For quantification of immunodetections, integrated intensities were corrected by background subtraction and normalized to untreated control cell phosphorylation of each signaling molecule duplicates in PathScan analyses. Relative mRNA expression was measured by cDNA amplification quantified with ViiA7 RUO software for real time PCR system version 1.2 in comparison to standard c-DNA dilution for amplification. Data were evaluated by analysis of variance (ANOVA) including Levene statistic as test of homogeneity of variances for the choice of Post Hoc Tests (Bonferroni for equal and Dunnett T3 for unequal variances) or by 2-tailed unpaired student's T Tests (using IBM SPSS statistics version 20), with significance accepted at values of p<0.05. EC50 or IC50 were calculated from sigmoidal dose-response (variable slope) by nonlinear regression of transformed data (GraphPad Prism 6).

## Results

### FOXO1 translocation in U-2 OS cells stably transfected with pEGFP-FOXO1

**Figure 1:**
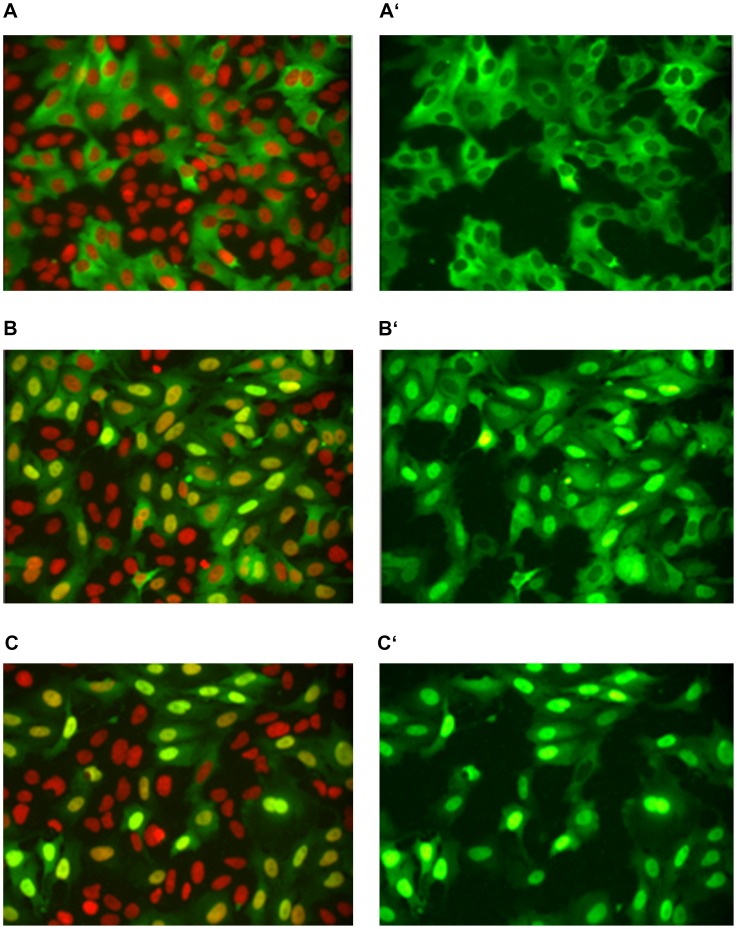
FOXO1-GFP translocation in stably transfected U-2 OS (human osteosarcoma cells). Intracellular localization of the forkhead box transcription factor O1 labelled with green fluorescent protein FOXO1-GFP visualized by fluorescence microscopy after nuclear staining with DAPI (colour coded red). Merged images in left panel: Cytoplasm green from FOXO1-GFP, nuclei red→orange→yellow→green depending on GFP overlay from FOXO1-GFP accumulated in nuclei (A–C). Right panel GFP images (A′–C′). (A, A′) Transfected U-2 OS cells with pEGFP-FOXO1 (after 1 h starvation in DMEM without FBS) treated with DMSO 0.15% (control) 2 h with cytoplasmic and perinuclear localization of FOXO1-GFP. (B, B′) Apigenin 30 µM in 0.15% DMSO induced nuclear accumulation of FOXO1-GFP. (C, C′) Luteolin 30 µM in DMSO 0.15% induced translocation of FOXO1-GFP from cytoplasm into nuclei in nearly all transfected U-2 OS cells with stable expression of GFP tagged FOXO1.

In analyses of micronutrients screened in concentrations of 1, 10 and 100 µM with our FOXO1 translocation assay, we calculated specific FOXO1 translocation factors as ratios FOXO1-GFP Nuc/Cyt after 2 h-treatment of U-2 OS normalized to DMSO mock treated cells. High FOXO1-import factors were obtained for each 100 µM of different polyphenols such as luteolin (3.1), apigenin (2.6), isokaempferide (2.3), kaempferol (2.2), quercetin (2.0), and resveratrol (1.6). For the most potent flavones luteolin and apigenin, induction of intranuclear accumulations of FOXO1-GFP is shown in our stably transfected U-2 OS after 2 h treatment with each 30 µM (Fig. 1A–C).

### Dose response and reversibility of FOXO1 translocation by insulin

**Figure 2:**
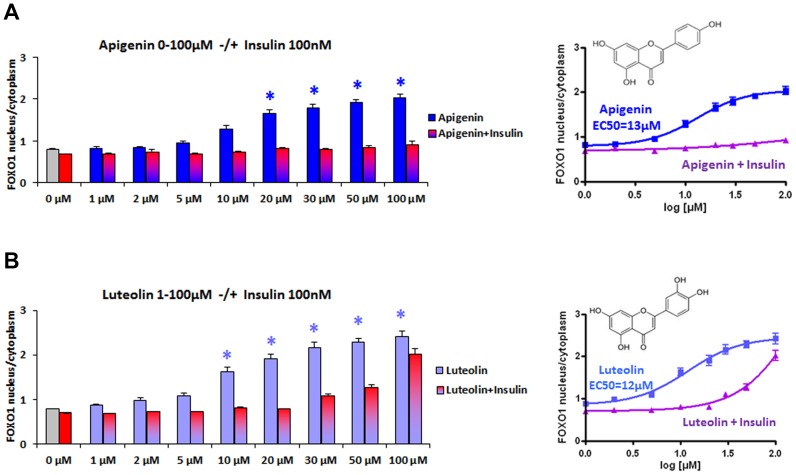
Dose-dependent induction of FOXO-GFP translocation by apigenin and luteolin, competition by insulin. Stably transfected human osteosarcoma cells with FOXO1-GFP (U2OS-FOXO1-GFP) treated with apigenin (A), luteolin (B) 1–100 µM for 2 h −/+ addition of insulin 100 nM after 30 minutes. Cells were fixed and stained with DAPI. Fluorescence microscopic detection of nuclei, segmentation of cells, quantification of GFP intensities measured in nuclear and cytoplasmic areas and calculation of the GFP-ratio nucleus/cytoplasm were performed for all FOXO-GFP expressing cells by BD Image Data Explorer. Nonlinear regression was performed with Graph Pad Prism. Results are presented as mean ± SEM of quadruplets with *(p<0.05) significant differences vs. control (ANOVA + Post Hoc Tests). (A) Dose-dependent accumulation of FOXO1 in nuclei induced by apigenin 1–100 µM shown as mean ratio of FOXO1 nucleus/cytoplasm + SEM (n = 4) *p<0.05 (Dunnett T3) and EC50 = 13 µM calculated by nonlinear regression from sigmoidal dose response. Reversion by insulin 100 nM induced FOXO1 translocation from nuclei into the cytoplasm. (B) Luteolin induced FOXO1 nuclear accumulation shown as mean ratio of FOXO1 nucleus/cytoplasm + SEM (n = 4) *p<0.05 (Bonferroni) with EC50 = 12 µM and competing insulin effect.

FOXO1 translocation was induced dose-dependently by apigenin (Fig. 2A) and luteolin (Fig. 2B). Insulin 100 nM was able to reverse FOXO1 nuclear accumulation induced by apigenin 1–50 µM nearly completely, when applied 30′ following apigenin treatment (Fig. 2A). FOXO1 accumulation induced by luteolin 30–100 µM was only partially reversible by insulin 100 nM at higher concentrations above 20 µM (Fig. 2B). Our results show the reversibility of FOXO1 translocation by both flavones in the presence of insulin to different degrees depending on one additional hydroxyl group for luteolin in position 3′ of ring B of the flavonoid structure. Kinetics of FOXO1 translocation

**Figure 3:**
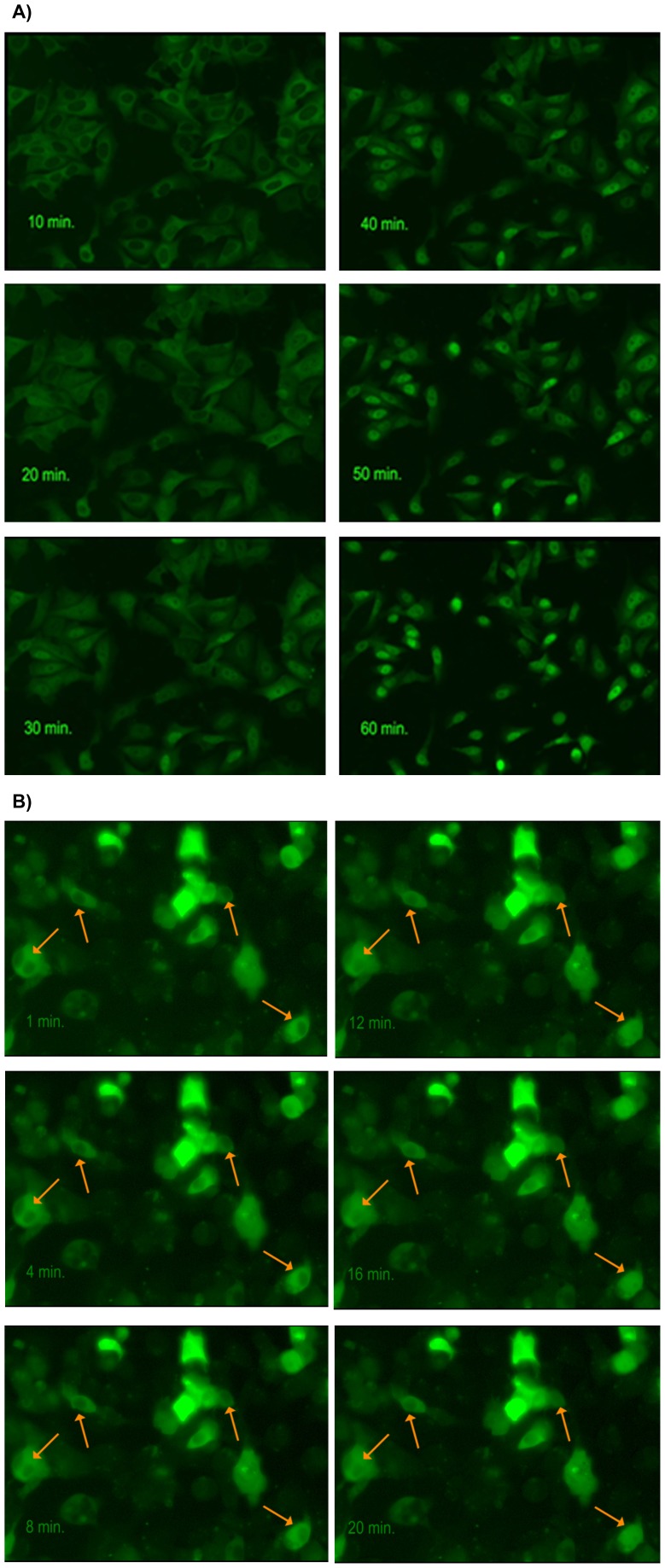
Time-dependent induction of FOXO-GFP translocation by apigenin A) and luteolin B) obtained by fluorescence-microscopic life cell imaging. Stably transfected human osteosarcoma cells with FOXO1-GFP (U2OS-FOXO1-GFP) were incubated with apigenin 10 µM (A) and transiently transfected human hepatic cells with FOXO1-GFP (HepG2-FOXO1-GFP) were incubated with luteolin 10 µM (B), respectively, for 1 h at 37°C in an incubation chamber connected to the inverted fluorescence microscope Axio Observer.Z1 from Zeiss. Images were taken every minute with the filter for GFP up to 60 minutes. FOXO1-GFP translocation with nuclear accumulation of FOXO1 is shown in a time-dependent course.

**Figure 4:**
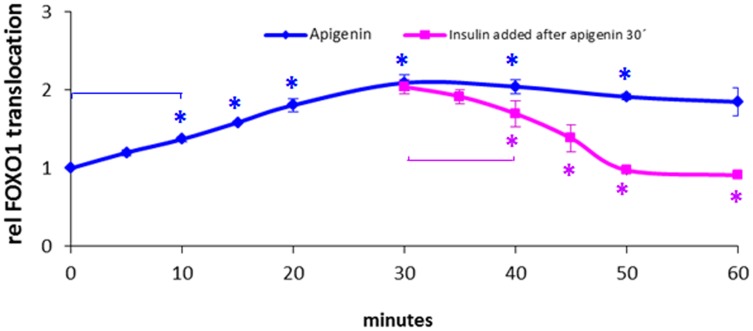
Time-dependent FOXO-GFP translocation induced by apigenin and reversed by insulin. Stably transfected human osteosarcoma cells with FOXO1-GFP (U2OS-FOXO1-GFP) were treated with apigenin 10 µM up to 1 h −/+ addition of insulin 100 nM after 30 minutes. Cells were fixed at indicated time points. GFP-ratio nucleus/cytoplasm was normalized to control at 0 minutes. Apigenin induced a significant FOXO1 nuclear translocation within 5–60 minutes of stimulation with maximal nuclear accumulation after 30 minutes. This accumulation was completely reversed by insulin during an incubation period from 30–60 minutes by competing export from nuclei into cytoplasm induced via the insulin signaling cascade. Experiments were performed in quadruplicates for each time interval and treatment condition. Cells were fixed and stained with DAPI for defining nuclear areas. Fluorescence microscopic analyses were performed in BD Pathway 435 system with BD Attovision using segmentation of cells by Cyto-Nuc Ring Band, quantification of GFP intensities measured in nuclear and cytoplasmic areas. The calculation of the GFP-ratios nucleus/cytoplasm were performed by BD Image Data Explorer. Results are presented as means of ratios normalized to control (0 minutes) ± SEM of 3 independent treatments. Significances versus control are shown for apigenin and insulin induced time dependent translocation of FOXO1 analyzed by Oneway ANOVA with Post Hoc Dunnett T3 and Bonferroni, respectively *(p<0.05).

Time-dependent FOXO1 translocation was shown by life cell imaging of U-2 OS and Hep G2. FOXO1-GFP translocation was observed in living cells during incubation for 1 h with apigenin, luteolin, and resveratrol and is shown for apigenin (U-2 OS) and luteolin (Hep G2) 10 µM (Fig. 3A and B, respectively). Apigenin 10 µM induced the highest nuclear accumulation with fast kinetics from 5′–30′ following application. Addition of insulin 100 nM at this time point of 30′ resulted in a complete export of FOXO1 during the following 20′ to the level before apigenin application (Fig. 4). Similar kinetics were observed with luteolin and resveratrol although differing in a less complete insulin reversibility (see Fig. 2B for less reversibility shown in dose response for luteolin).

### Role of ROS in triggering apigenin- and luteolin induced FOXO1 translocation

**Figure 5:**
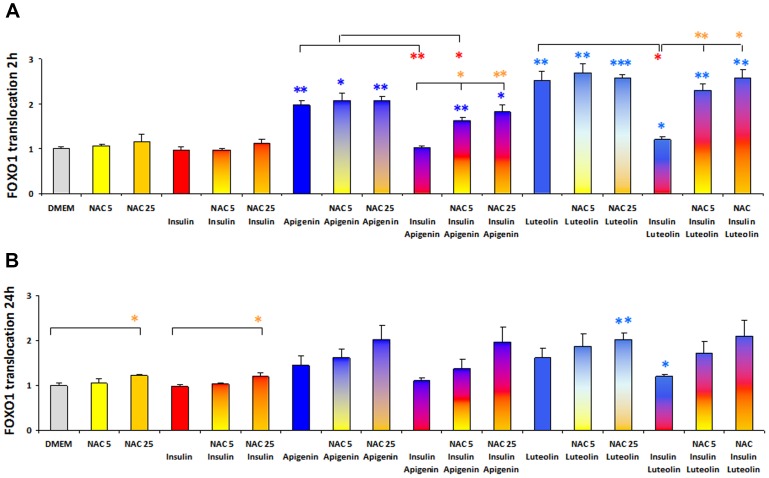
FOXO1-GFP translocation induced by apigenin and luteolin in the presence of N-acetyl-L-cysteine, reduced reversion by insulin. Stably transfected human osteosarcoma cells with FOXO1-GFP (U2OS-FOXO1-GFP) were incubated with the antioxidant N-acetyl-L-cysteine (NAC) 5 mM and 25 mM for 30 minutes before treatment with apigenin 30 µM and luteolin 30 µM −/+ insulin 100 nM for 2 h and 24 h respectively. Cells were fixed and stained with DAPI. Experiments were performed in quadruplets and fluorescence microscopic analyses performed with the BD Pathway 435 system, BD Attovision and BD Image Data Explorer. GFP-ratios nucleus/cytoplasm were normalized to untreated control cells. Results are presented as relative FOXO1 translocations resulting from means of ratios GFP Nuc/Cyt normalized to control ± SEM. Oneway ANOVA and Dunnett T3 significances are shown *(p<0.05), **(p<0.01), and ***(p<0.001) in blue for flavone induced FOXO1 nuclear accumulation vs control DMEM, in red for insulin induced FOXO1 export into cytoplasm, and in yellow for NAC effect on FOXO1 translocation.

Analyses were performed in the presence of antioxidants to test whether flavone induced FOXO1 translocation could be dependent on oxidative stress. After preincubation of U-2 OS cells with the antioxidant N-acetyl-L-cysteine (NAC) 5 mM and 25 mM for 30′, the induction of FOXO1-GFP translocation by apigenin 30 µM during 2 h incubation was not disturbed resulting in a 2-fold accumulation in nuclei. Unexpectedly, NAC restricted the competing export of FOXO1 by insulin 100 nM in a dose dependent manner (Fig. 5A). The higher import-factor of 2.5 induced by luteolin 30 µM was reduced by insulin to 1.2. In the presence of NAC 5 mM the nuclear accumulation factor was reduced slightly to 2.3 and NAC 25 mM abolished the insulin effect (Fig. 5A). These results show that apigenin and luteolin did not induce FOXO1 import into nuclei by provoking oxidative stress while the insulin induced export of FOXO1 depended on the oxidative status of the cell and was reduced by increasing antioxidants.

Treatment for 24 h with apigenin 30 µM showed a lower rate of FOXO1 nuclear accumulation of 1.5-fold, which was enhanced after pretreatment with NAC 5 mM to 1.6-fold and with NAC 25 mM to 2-fold accumulation (Fig. 5B). During 24 h of incubation, FOXO1 seemed to undergo an equilibrium between nuclear import and export, and the 2-fold nuclear accumulation seen after 2 h could be maintained only in the presence of NAC 25 mM, which was able to reduce FOXO1 export induced by insulin as well. The reduction of apigenin induced FOXO1 accumulation by insulin was not complete after 24 h, diminished in the presence of NAC 5 mM and abolished with NAC 25 mM (Fig. 5B). 24 h luteolin 30 µM resulted in a 1.6-fold FOXO import which increased to 1.9- and 2-fold in the presence of NAC 5 mM and 25 mM respectively, the latter not being reduced by insulin (Fig. 5B). These data show that the insulin induced FOXO1 translocation from the nucleus to the cytoplasm was disturbed by prevention of oxidative stress and that the flavone induced nuclear accumulation was not dependent on reactive oxygen species (ROS).

### Cell viability

Cell viability assays with apigenin and luteolin 1–100 µM were performed in insulin sensitive HepG2 cells expressing endogenous FOXO1, which were used for gene expression analyses. Using the cell proliferation assay from Promega, we found significant reductions of cell viability after 24 h only about 20% for apigenin 100 µM and 33% for luteolin 100 µM. Up to 50 µM of both flavones cell survival rates were not affected (Table 2). With resveratrol similar survival rates were obtained (data not shown).

**Table 2:** Cell proliferation assay.

	Cell vitality [%] vs control [0 µM] ± SEM (n = 3)	
flavone-concentration	Apigenin	Luteolin
0 µM	100.00±1.40	100.00±1.40
1 µM	102.65±1.36	103.88±1.72
2 µM	99.70±1.02	100.35±1.03
5 µM	93.84±2.36	97.29±1.86
10 µM	89.28±4.56	87.80±1.34
20 µM	83.65±1.50	102.62±1.20
50 µM	82.08±2.57	98.67±0.58
100 µM	*79.51±1.80	***66.92±1.69
Oneway ANOVA	p = 0.000	p = 0.000
Levene statistic	p = 0.029	p = 0.159
	*p<0.05 Dunnett T3	***p<0.001 Bonferroni

Vitality of HepG2 cells treated with apigenin and luteolin 1 µM–100 µM for 24 h.

HepG2 survival was measured using the CellTiter96 Aqueous ONE Solution from Promega applied to cell-cultures after 24 h treatment with flavones apigenin and luteolin in the range from 1–100 µM. Following incubation with 20 µM (3-(4,5-dimethylthiazol-2-yl)-5-(3-carboxymethoxy phenyl)-2-(4-sulfophenyl)-2H-tetrazolium inner salt (MTS)/phenazine methosulfate (PMS - electron coupling reagent) for 4 h at 37°C, the optical density of the MTS bioreduction product formazan was measured at 490 nm. Means of OD-values were normalized to mock treated cells (100% survival) and analyzed by Oneway ANOVA for 0, 1, 2, 5, 10, 20, 50, and 100 µM apigenin and luteolin respectively with Levene statistics for analyses of variance. Significant reductions of vitality were found for 100 µM apigenin or luteolin vs 0 µM in mock treated control cells with DMSO 0.5% analyzed by Dunnett T3 (unequal variance for apigenin) or Bonferroni (equal variance for luteolin) respectively.

### Gene expression

Profiling of gene-expression was performed in human hepatoma cells HepG2 in order to obtain data for the gluconeogenic enzymes PEPCK and G6Pc expressed in liver as well as lipogenic enzymes FASN and ACC. HepG2 cells were treated 2 h and 24 h with apigenin and luteolin, RNA extracted and reverse transcribed for qRT-PCR with specific primer-pairs shown in table 1. Gene regulation was calculated as ratio of mRNA expression normalized to RPL32 after treatment with specific substances versus mock treated cells with the substance solvent.

### Gluconeogenic gene regulation

**Figure 6:**
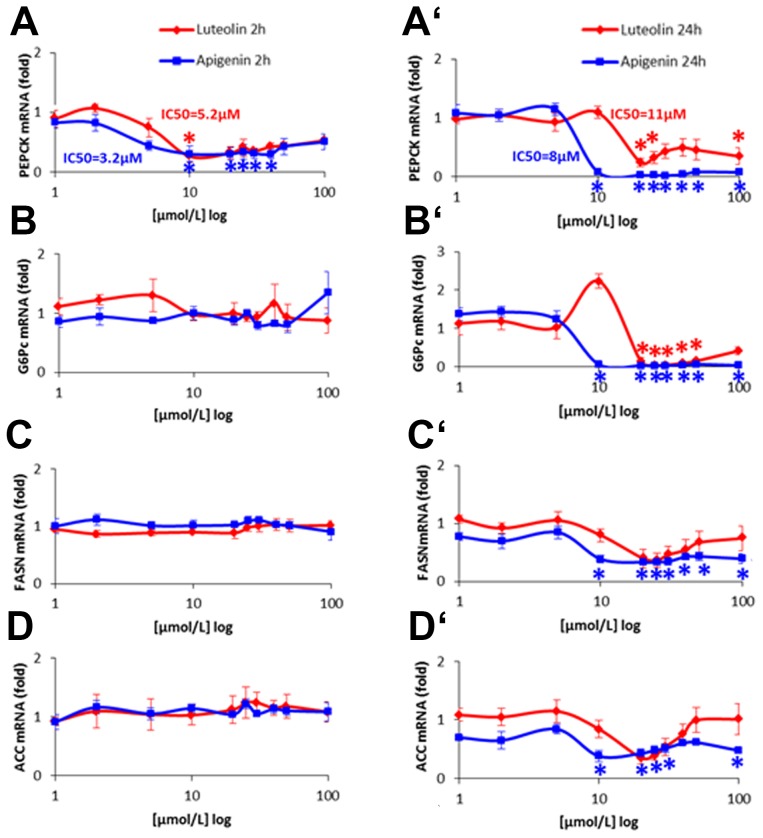
Time- and dose-dependent modulation of gene expression in HepG2 cells induced by apigenin and luteolin. A–D: Human hepatoma cells (HepG2) were cultivated in EMEM + 10% FBS and starved without FBS 16 h before stimulation. Apigenin and luteolin were applied in the range of 1–100 µM diluted in EMEM. Incubation of HepG2 was performed for 2 h and 24 h respectively. Total RNA was extracted with Nucleospin RNA II isolation kit and reverse transcribed with the High capacity cDNA reverse transcription kit for quantitative realtime PCR (qRT-PCR) in triplicates using the Power SYBR green PCR master mix with primers pairs described in Table 1. qRT-PCR was run in triplicates using cDNA from control cells treated with DMSO 0.5% for standard dilutions. Levels of mRNA were normalized to the houskeeping gene ribosomal protein (RPL32). Three independent experiments were performed with different passages of HepG2. Results are presented as fold mRNA expression normalized to control expression as means ± SEM and significances versus control *(p<0.05). Gluconeogenic (A) phosphoenolpyruvate carboxykinase (PEPCK) and (B) glucose-6-phosphatase (G6Pc), lipogenic (C) fatty-acid synthase (FASN) and (D) acetyl-CoA-carboxylase (ACC).

**Figure 7:**
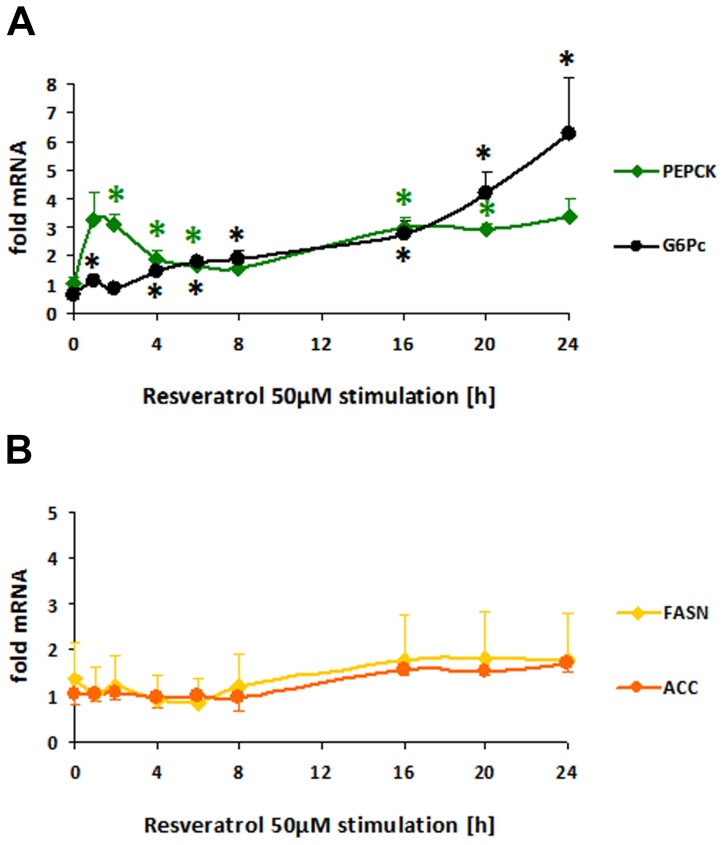
FOXO targetgene-expression in HepG2 (human hepatoma) cells modulated by polyphenolic resveratrol in a time-dependent course. A–B: HepG2 cell cultures grown in EMEM + FBS 10% and starved for 16 h without FBS were stimulated with resveratrol 50 µM in 0.125% DMSO in EMEM for 1–24 h. RNA was extracted with Nucleospin RNA II isolation kit and reverse transcribed with the High capacity cDNA reverse transcription kit for quantitative realtime PCR (qRT-PCR) in triplicates using the Power SYBR green PCR master mix with primers pairs described in Table 1. Modulated mRNA levels normalized to ribosomal protein (RPL32) housekeeping gene are shown as fold mRNA of basal expression in mock stimulated HepG2 means ± SEM (n = 3) of 3 independent experiments with different passages of HepG2 with significances (t-test) versus DMSO-control *p<0.05. (A) gluconeogenic phosphoenolpyruvate carboxykinase (PEPCK) up-regulated by resveratrol in a biphasic manner (1–4 h and 16–24 h) and glucose-6-phosphatase (G6Pc) with continuous increase (4–24 h), (B) lipogenic fatty-acid synthase (FASN) and acetyl-CoA-carboxylase (ACC) slight induction 8–24 h and 16–24 h respectively.

PEPCK mRNA was down-regulated by flavones in a dose dependent manner after 2 h with an IC50 of 3.2 µM for apigenin and 5.2 µM for luteolin (Fig. 6A). After 24 h the effect of reduction was stronger for apigenin 10–100 µM resulting in a nearly complete suppression, with a slightly elevated IC50 of 8 µM while luteolin was less effective with an IC50 of 11 µM (Fig. 6A′). Reduction of G6Pc mRNA could not be detected after 2 h (Fig. 6B), but after 24 h a complete down-regulation of G6Pc mRNA was achieved with apigenin 10–100 µM and luteolin 20–50 µM, while 10 µM luteolin induced the G6Pc mRNA transcription (Fig. 6B′). In contrast we found an up-regulation of PEPCK and G6Pase gene expression by the polyphenol resveratrol 50 µM in a time-dependent course (Fig. 7A). PEPCK was induced in a biphasic manner (1–4 h and 16–24 h) possibly by SIRT1 activation of FOXO1 and a second phase induction activated by autofeedback regulation of SIRT1 expression via FoxO1 as *described by Xiong et al.* [*26*] *while G6Pc mRNA increased continuously (4–24 h).*

### Lipogenic gene regulation

Treatment for 24 h with apigenin 10–100 µM reduced ACC mRNA about 50% and FASN mRNA to 40–50% (Fig. 6C′–D′) while treatment for 2 h had no effect (Fig. 6C–D). Luteolin elicited similar effects between 20–30 µM (Fig. 6C′–D′). By contrast, slight up-regulations of FASN and ACC were found 8–24 h after application of resveratrol 50 µM (Fig. 7B).

### Knockdown of modulating factors by siRNA transfection

For the investigation of mechanisms behind these differential patterns of gene expression, we knocked down the transcription factors FOXO1, FOXO3a which are known to induce PEPCK and G6Pc and NRF2 as a potential modulator, and AKT as a FOXO-inhibitor and SIRT1 deacetylase as a FOXO-activator. HepG2 cells were transfected with siRNAs 48 h before treatment with apigenin and luteolin each 20 µM or DMSO 0.1% for 24 h followed by extraction of RNA for quantitative RT-PCR. The knockdown effects of each set of siRNAs normalized to NonTarget-siRNA are summarized in Table 3 shown as percentage of each mRNA expression ± SEM (n≥4).

**Table 3:** siRNA-knockdown.

si RNAs	FOXO1 mRNA	FOXO3a mRNA	SIRT1 mRNA	AKT mRNA	NRF2 mRNA
**NonTarget control**	100.00±7.10	100.00±4.98	100.00±4.13	100.00±3.84	100.00±4.74
**FOXO1**	**48.49±3.73	91.21±5.81	111.00±9.43	94.71±6.64	109.88±7.57
**FOXO3a**	97.18±14.17	***25.85±4.87	114.22±10.72	89.08±9.15	112.30±8.52
**SIRT1**	103.18±9.36	100.20±4.44	*50.70±10.33	90.33±5.85	116.83±14.49
**AKT**	95.23±16.98	106.22±18.20	112.00±10.50	**36.08±6.68	116.50±8.64
**NRF2**	94.75±10.34	101.00±11.87	108.10±5.40	101.70±8.46	**50.76±3.89
**FOXO1+FOXO3a**	*55.15±5.92	***38.50±3.79	120.35±14.42	101.38±8.93	121.98±12.45
**FOXO1+SIRT1**	**63.80±5.99**	101.10±0.86	*51.23±4.58	97.63±5.77	129.98±11.00
**FOXO1+AKT**	**61.67±8.53**	86.85±8.72	104.40±8.25	*41.72±7.36	95.30±8.28
**FOXO1+NRF2**	**65.57±6.53**	95.65±10.70	101.65±10.33	88.78±10.37	**52.60±5.17
**FOXO3a+SIRT1**	99.20±2.19	***41.48±4.07	*53.98±5.37	91.13±2.19	112.25±4.88
**NRF2+AKT**	84.43±11.25	103.23±24.09	94.60±2.25	***47.92±3.90	***55.30±2.63
Oneway ANOVA	p = 0.000	p = 0.000	p = 0.000	p = 0.000	p = 0.000
Levene statistic	p = 0.027	p = 0.002	p = 0.100	p = 0.030	p = 0.022
	* P<0.05 Dunnett T3 **p<0.01 Dunnett T3	***p<0.001 Dunnett T3	* p<0.05 Bonferroni	* P<0.05 Dunnett T3 **p<0.01 Dunnett T3 ***p<0.001 Dunnett T3	**p<0.01 Dunnett T3 ***p<0.001 Dunnett T3

Values: mRNA expression mean [%] ± SEM of NonTarget control (n≥4).

Knockdown by specific siRNA pools transfected with D4 (Dharmacon transfection reagent) in HepG2 cells. Single knockdowns were performed for FOXO1, FOXO3a, SIRT1, AKT, or NRF2 (n = 8) and double knockdowns for combinations FOXO1/FOXO3a, FOXO1/SIRT1, FOXO1/AKT, FOXO1/NRF2, FOXO3a/Sirt1 or NRF2/AKT (n = 4) using each half of the amount of siRNA used in transfection-mixes. 48 h after transfection of cells including a starvation period of 16 h in EMEM without FBS, HepG2 cells were treated with DMSO 0.5% for 24 h (mock stimulation), RNA extracted, reverse transcribed and qRT-PCR performed with primers given in table 1. Quantified relative amplification vs housekeeping gene RPL32 expression was normalized to the rate in HepG2 transfected with non-target NT siRNA (100% rate of expression).

Gene expression profiling was performed in the presence of non-targeting NT siRNA transfection, with 5 specific single and 6 combined double knockdowns. The effects of apigenin and luteolin were measured versus DMSO mock stimulation in HepG2 cells after 12 different siRNA transfection conditions each. The next section reports our results on i) Effects of siRNAs on gene expression and ii) Flavone effects upon Non Target and specific siRNA knockdowns.

### PEPCK

**Figure 8:**
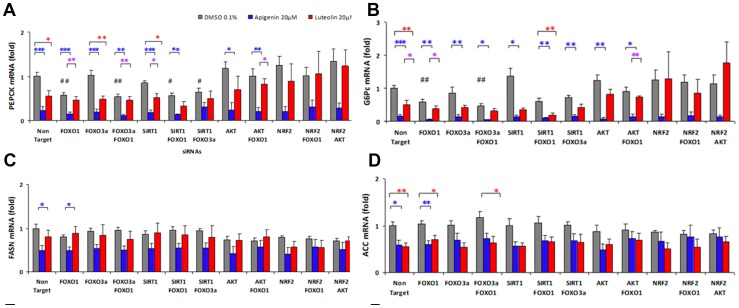
Gene expression profiling upon siRNA knockdowns and analysis of modulations by apigenin and luteolin. A–D: Subconfluent human hepatoma cells (HepG2) were transfected with silencing RNA (siRNA) for forkhead box transcription factor O1 (FOXO1), forkhead box transcription factor O3a (FOXO3a), sirtuin1 (SIRT1), protein kinase B (PKB/AKT), nuclear factor (erythroid-derived2)-like2 (NRF2) and non targeting (NT)-siRNA with DharmaFECT4 in EMEM + 10% FBS for 48 h including a starvation period without FBS of 16 h preceeding stimulation with apigenin and luteolin each 20 µM for 24 h. RNA was extracted, reverse transcribed and cDNA from control cells after treatment with DMSO 0.1% were used for standard dilutions. For 14 targets qRT-PCR was run with SYBR green in triplicates. Levels of mRNA were normalized to the expression of houskeeping ribosomal protein (RPL32) mRNA. At least four experiments (n = 4–8) transfecting each siRNA or combined siRNAs for single and double knockdowns and control transfections with NT-siRNA followed by apigenin, luteolin or mock treatment with DMSO 0.1% were performed with different passages of HepG2. Ratios of mRNA levels vs basal expression in NT-siRNA transfected cells were calculated for knockdown induced fold mRNA of basal levels (grey columns). T-tests were performed for independent samples and significances versus control are shown ^#^p<0.05, ^##^ p<0.01 and ^###^p<0.001 according to Levene statistics for equality of variances with corrections for equal variances. Expression profiles are shown for DMSO mock stimulation (grey) apigenin 20 µM (blue) and luteolin 20 µM (red) + SEM and significant differences upon each knockdown condition obtained by Oneway ANOVA and posthoc tests with multiple comparisons Dunnet T3 for Levene unequal or Bonferroni for Levene equal variances indicated as *p<0.05, **p<0.01 and ***p<0.001 (blue apigenin vs DMSO, red luteolin vs DMSO, purple apigenin vs luteolin). (A) phosphoenolpyruvate carboxykinase (PEPCK)-, (B) glucose-6-phosphatase (G6Pc)-, (C) fatty-acid synthase (FASN)-, (D) acetyl-CoA carboxylase (ACC).

**i) Effects of siRNAs on gene expression:** PEPCK mRNA levels were reduced significantly upon knockdowns of FOXO1 (p = 0.002), FOXO1/FOXO3a (p = 0.009), and FOXO1/SIRT1 (p = 0.013) indicating a clear dependence of the PEPCK expression on the transcription factor FOXO1. These effects may be explained by a FOXO1-dependent expression of PEPCK. The reduction of PEPCK mRNA was abolished in combined knockdowns of FOXO1/AKT while AKT single knockdowns resulted in slightly elevated expression. This underlines the role of FOXO1 which would be less inactivated by phosphorylation at serine 256 upon AKT knockdown. Knockdowns of NRF2/AKT and NRF2 induced the PEPCK expression slightly and NRF2/FOXO1 resulted in unchanged PEPCK mRNA levels thus preventing the reduction observed upon FOXO1 knockdown (Fig. 8A).

**ii) Flavone effects upon Non Target and specific siRNA knockdowns:** Apigenin 20 µM suppressed PEPCK mRNA significantly (p<0.001) after 24 h in NT siRNA transfected HepG2 controls. Significant reductions by apigenin were found also upon knockdowns of FOXO1 (p<0.001), FOXO3a (p<0.001), FOXO1/FOXO3 (p = 0.002), SIRT1 (p<0.001), FOXO1/SIRT1 (p = 0.003), AKT (p = 0.04), and FOXO1/AKT (p = 0.005). This indicated that the apigenin induced down-regulation of the PEPCK expression did not depend on FOXO1, FOXO3a, SIRT1, and AKT. Upon NRF2-knockdown and combinations NRF2/FOXO1 and NRF2/AKT significances were lost although the apigenin induced suppression was still apparent. Apigenin obviously exerts quite powerful effects and the loss of significance indicates an involvement of NRF2, FOXO1 and AKT related pathways.

Luteolin 20 µM reduced PEPCK mRNA levels significantly (p = 0.024) but less effectively than apigenin with significant differences between both flavones after knockdown of FOXO1 (p = 0.004), FOXO1/FOXO3 (p =  0.009), SIRT1 (p = 0.022), and FOXO1/AKT (p = 0.024). Luteolin down-regulated PEPCK in the presence of FOXO3a (p = 0.005) and SIRT1 (p = 0.023) siRNAs. Knockdowns of FOXO1, FOXO1/FOXO3a, FOXO1/SIRT1, FOXO3a/SIRT1 abolished the inhibition by luteolin. In the presence of NRF2 siRNA or combined NRF2/FOXO1 and NRF2/AKT siRNAs luteolin did not show any down-regulating effect. This indicated potential roles of FOXO1 and NRF2 in mediating the action of luteolin. It also supports the conclusion that NRF2 inhibits PEPCK expression as observed upon NRF2 knockdown. Apigenin was clearly more potent than luteolin as observed in the dose response curves for inhibition of PEPCK before (Fig. 6A). However, due to the high potency of apigenin, the knock down results were less clear than for luteolin. We interpret this as a consequence of the incomplete knockdown using the siRNA technique. The remaining FOXO1 appears to be sufficient to mediate the apigenin induced suppression of PEPCK. The roles of FOXO3a and SIRT1 appear to be minor, however, the combined knockdown prevented the inhibition by both flavones, which may indicate a limited role.

### G6Pc

**i) Effects of siRNAs on gene expression:** G6Pc mRNA levels were reduced significantly upon knockdowns of FOXO1 (p = 0.004) and FOXO1/FOXO3a (p = 0.003). The single knockdown of FOXO3a had no effect, exhibiting a clear dependence on the transcription factor FOXO1 with minor contribution of FOXO3a. The FOXO1 dependent reduction of G6Pc mRNA was nearly abolished in combined knockdowns of FOXO1/AKT and upon a single knockdown of AKT the expression was slightly elevated as may be expected due to the inactivation of FOXO1 and FOXO3a via AKT. Knockdowns of NRF2 either alone or in combination with AKT or FOXO1 slightly induced the expression of G6Pc (Fig. 8B). Therefore deficiency of NRF2 promotes the transcription of G6Pc as well as PEPCK indicating similar regulation of both gluconeogenic genes.

**ii) Flavone effects upon Non Target and specific siRNA knockdowns:** Apigenin 20 µM suppressed G6Pc mRNA significantly (p<0.001) after control NT siRNA transfection. Significant reductions were also observed in the presence of all siRNAs while knockdown of NRF2 prevented a significant inhibition of G6Pc by apigenin (Fig. 8B). Luteolin 20 µM reduced G6Pc mRNA significantly after transfection of NT siRNA (p = 0.003), SIRT1 siRNA (p = 0.047) and SIRT1/FOXO1 siRNAs (p = 0.008). However, any combination including NRF2 prevented the down-regulation of G6Pc by luteolin indicating a significant role of NRF2. The down-regulation of G6Pc by luteolin was less effective than by apigenin differing significantly in NT (p = 0.035), upon FOXO1- (p = 0.022) and FOXO1/AKT-knockdowns (p = 0.004). These differences possibly depend on the lower potency of luteolin than of apigenin for the down-regulation of G6Pc (see figure 6B dose response).

#### FASN

FASN mRNA levels were reduced slightly but not significantly after transfection of FOXO1-, AKT-, NRF2-, AKT/FOXO-, NRF2/FOXO-, and NRF2/AKT-siRNAs versus NT-siRNA (Fig. 8C). Apigenin 20 µM reduced FASN mRNA levels after NT siRNA (p = 0.021) and after FOXO1 siRNA (p = 0.012). This reduction was abolished in double knockdowns of AKT/FOXO1, NRF2/FOXO1, and NRF2/AKT indicating some contribution of AKT and NRF2 to the down-regulation of FASN by apigenin (Fig. 8C). NRF2 again appears to be involved in the inhibitory effect of apigenin on metabolic enzyme expression. For luteolin 20 µM significant down-regulations were not detected in siRNA transfected HepG2 cells in contrast to data in untransfected cells (see figure 6C′).

#### ACC

ACC mRNA levels were not significantly affected by any of the knockdowns (Fig. 8D). Only slight reductions were observed following AKT-, NRF2-, and combined siRNA transfections similar to FASN-mRNA levels. Apigenin 20 µM and luteolin 20 µM reduced ACC mRNA levels significantly in NT siRNA control (p = 0.014 and p = 0.008 respectively) and after FOXO1 siRNA (p = 0.003 and p = 0.023). Upon the double knockdown of FOXO1/FOXO3a only the down-regulation by luteolin was significant (p = 0.039). The efficiencies of apigenin and luteolin induced ACC regulations did not differ significantly (Fig. 8D).

### Effects of flavones on intracellular signaling pathways

For analyses of the influence of flavones on the phosphorylation status of proteins, HepG2 cells were treated after 16 h starvation with apigenin 20 µM, luteolin 20 µM or DMSO 0.1% for mock stimulation for 30′ with and without preincubation for 15′ with insulin 100 nM. Analyses were performed for phosphorylated AKT(Thr308), AKT(Ser473), PRAS40(Thr246), mTOR(Ser2448), p70S6K(Thr389), and in duplicates.

**Figure 9:**
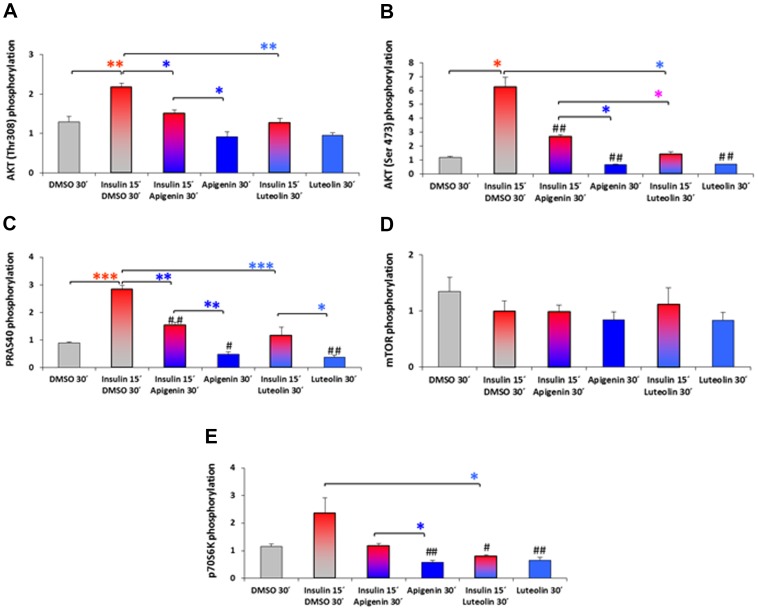
Posttranslational phosphorylation in the AKT-signaling cascade. A–E: Modifications of molecules at nodal points of AKT intracellular signaling were analysed in lysates of human hepatoma (HepG2) cells treated with apigenin 20 µM, luteolin 20 µM or DMSO 0.1% for 30′ ± pretreatment with insulin 100 nM for 15′. Cell lysis was performed in presence of phosphatase inhibitors and lysates analyzed for protein phosphorylation or cleavage using the PathScan Intracellular Signaling Array Kit (fluorescent readout) from Cell Signaling technology. Data are shown as means of integrated intensities of three independent experiments (n = 3) + SEM normalized to untreated control cells and significances shown as # p<0.05 and ## p<0.01 vs mock stimulated HepG2 with DMSO 0.1% (T-Test) or * p<0.05, ** p<0.01 and ***p<0.001 as indicated (Oneway ANOVA and posthoc Bonferroni or Dunnett T3 multiple comparisons). (A) protein kinase B PKB/AKT (Thr308), (B) AKT (Ser473), (C) proline-rich AKT/PKB substrate 40 kDa PRAS40 (Thr246), (D) mammalian target of rapamycin mTor (Ser2448), (E) p70S6 kinase p70S6K (Thr389), (F) ribosomal protein S6 (Ser235/236).

Phosphorylation of AKT at threonine 308 and serine 473 was significantly induced by insulin 100 nM. Both flavones apigenin 20 µM and luteolin 20 µM added 15′ after insulin reversed the AKT phosphorylation during the following 30′ and reduced the basal phosphorylation of AKT (Fig. 9A+B).

The proline-rich AKT/PKB substrate 40 kDa (PRAS40) was phosphorylated at threonine 246 significantly by insulin relieving PRAS inhibition of mTOR in the complex mTORC1. Apigenin 20 µM and luteolin 20 µM reduced not only the insulin induced phosphorylation but also the basal phosphorylation status of PRAS40 (Fig. 9C). Regarding the mammalian target of rapamycin mTOR phosphorylation levels at serine 2448 we did not detect any significant modulations neither by insulin nor by the flavones apigenin or luteolin despite a slight reduction in all conditions versus DMSO mock stimulation (Fig. 9D).The phosphorylation of the p70S6 kinase at threonine 389 was induced by insulin 100 nM and reversed by apigenin 20 µM to the level without insulin and by luteolin reduced even to a lower level of p70S6K (Thr389) indicating a significantly stronger effect of luteolin (Fig. 9 E). The basal phosphorylation in mock stimulated HepG2 cells was significantly reduced upon apigenin and luteolin indicating dephosphorylating activities.

### IGF-1 receptor phosphorylation

**Figure 10:**
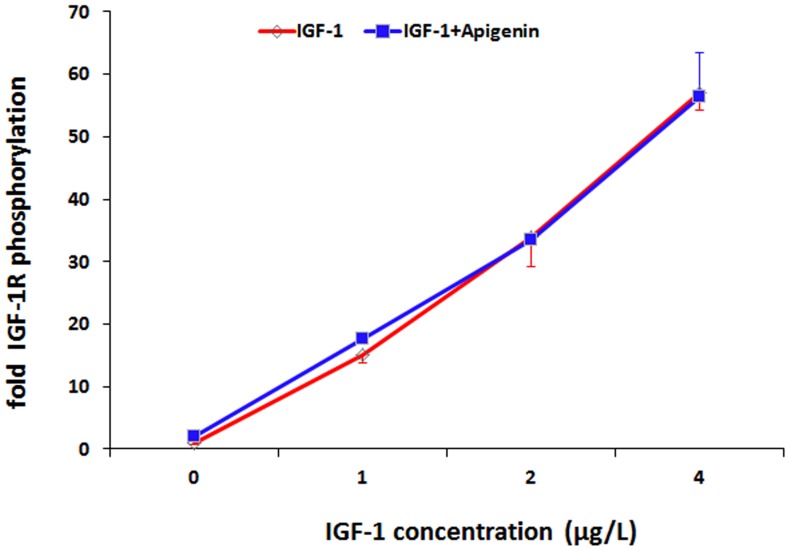
Effect of apigenin on the auto-phosphorylation of the IGF-1 receptor. Human embryonic kidney (HEK) cells overexpressing the insulin-like growth factor receptor (IGF-1R) were incubated with different concentrations of IGF-1 in the presence or absence of apigenin 20 µM for 16 minutes. Stimulated cells were lysed and transferred to a normal ELISA-sandwich assay using a mouse monoclonal IGF-1R antibody as a capture antibody and anti-mouse horseradish peroxidase-conjugated anti-phosphotyrosine monoclonal antibody as a detection antibody to quantify the phosphorylation of IGF-1 receptor. Bars show means ± SEM of two experiments performed in duplicate.

The dose-dependent phosphorylation of the IGF-1 receptor (IGF-1R) induced by IGF-1 in human embryonic kidney (HEK) cell cultures overexpressing IGF-1R analyzed with the kinase receptor activation assay (KIRA) was not influenced by apigenin 20 µM indicating a direct regulation of the PKB/AKT-signaling pathway distal to the IGF-1 receptor (Fig. 10).

## Discussion

FOXO1 is an important metabolic regulator which is inactivated by insulin. Physiologically it regulates metabolism in the fasting state and under caloric restriction when insulin levels are low [18]. FOXO transcription factors appear to orchestrate many of the beneficial responses to caloric restriction and may therefore represent an interesting target for improving metabolism in the presence of obesity and increased insulin levels. Polyphenols were proposed to mediate the protective effects of Mediterranean diets [11]. We therefore screened for plant derived polyphenols activating FOXO1 by use of a GFP-tagged FOXO1 screening assay. Remarkably, plant polyphenols contain many potent activators of FOXO1 and we further studied the mechanism of action of the two most potent activators identified, apigenin and luteolin. FOXO1 is known to induce hepatic glucose production by inducing the transcription of PEPCK and G6Pc which should be disadvantageous for prevention of T2DM. We therefore further focused on the regulation of the gluconeogenic and lipogenic enzymes by luteolin and apigenin.

Our results showed the potent and rapid translocation of FOXO1 by both flavones and its reversibility in the presence of insulin which is well explained by their inhibition of the AKT pathway. This inhibition is located somewhere upstream of AKT but below the level of the insulin/IGF-1 receptor. Since these effects occurred within minutes it implies that these flavones would mildly antagonize insulin actions upon food intake in a competitive manner. It also emphasizes that FOXO shuttling between nucleus and cytoplasm is a highly dynamic process. Polyphenol actions are often attributed to their antioxidative potential. We therefore examined whether the effects of the flavones were related to oxidative stress. This was obviously not the case since treatment with an antioxidant did not alter the ability of either flavone to translocate FOXO1. Remarkably, the activity of insulin to export FOXO1 was impaired in the presence of antioxidants corresponding to the well known regulation of redox mechanisms by insulin [27]. The inhibition of the AKT pathway by flavones has been previously described in HL60 and other cell types [28].

We further studied the regulation of gluconeogenic and lipogenic enzymes in the human hepatoma cell line HepG2. The polyphenol resveratrol which also induced nuclear translocation of FOXO1 induced the expression of PEPCK and G6Pc as expected. By contrast, the flavones inhibited the expression of PEPCK and G6Pc dose and time dependently. A down-regulation of gluconeogenic gene expression over a wide range of flavone concentrations is observed for the first time. Remarkably, PEPCK was reduced already after 2 h while the inhibition of PEPCK was apparent after 24 h indicating some differences in the mechanisms involved. The lipogenic enzymes FAS and ACC were also both down regulated by the flavones although with lower potency and a latency of 24 h.

In a next step we analyzed the role of the transcription factors FOXO1, FOXO3 and NRF2 in the regulation of the gluconeogenic enzymes and additionally the kinase AKT and the sirtuin deacetylase SIRT1 using siRNA technology to reduce their expression. The knock down of FOXO1 reduced the mRNA expression levels of PEPCK and G6Pc confirming its role in HepG2 cells. Neither FOXO3A, NRF2 nor SIRT1 or AKT knock down had significant effects on basal expression. We also tested double knock downs to search for interactions of the transcription factors. As expected, the combined knock down of FOXO1 and FOXO3a or FOXO1 and SIRT1 did not alter the inhibitory action of FOXO1 knockdown on PEPCK-expression. However, knock down of NRF2 completely prevented the effect of FOXO1 on the expression of PEPCK and G6Pc. To explain these NRF2 effects we analyzed the promoter sequence of human PEPCK (2000 bp of 5′UTR derived from NG_008205 for PCK1 on chromosome 20 analyzed by Genomatix Matinspector using the matrix family library for core/matrix similarity) and found 10 binding motifs for NRF2 (ARE Matrix family V$AP1R for MAF and AP1 related factors), the last one near to the transcription start site close to the FOXO1-binding site IRE2 described by Park et al. 2010 [29]. This suggests that NRF2 has potential direct effects on the expression of PEPCK and apparently antagonizes the inhibition by FOXO1. We postulate that a reduced availability of NRF2 (knockdown see Table 3) for binding to ARE could alleviate binding of FOXO to the IRE and thereby promote PEPCK transcription. PEPCK mRNA levels were lowered upon double knockdowns of FOXO3a/SIRT1 (p = 0.049) but FOXO3a alone did not show any effect and a faint reduction by SIRT1 knockdown failed to reach significance. Lower levels upon knockdown of SIRT1 indicate some contribution of nuclear deacetylase activity. Therefore a role of FOXO3a on PEPCK transcription could not be confirmed and some contribution of SIRT1 via intranuclear deacetylation appeared to enhance the PEPCK expression. Comparing the gluconeogenic gene expression of PEPCK and G6Pc, our findings suggested that FOXO1 was one important transcription factor for both and FOXO3a played a minor role. Higher levels of expression upon knockdowns of AKT underline the role of FOXOs with reduced inactivating phosphorylation capacity by AKT.

Lipogenic gene expressions for FASN and ACC in HepG2 did not depend on FOXO1, FOXO3a and SIRT1. Both mRNA levels were slightly reduced upon AKT- and NRF2 knockdown leading to the assumption of a partial dependence on the transcription factor NRF2.

We then tested the efficacy of the flavones in the presence of the knockdowns. The reduction of PEPCK- and G6Pc-mRNA by the flavones was not reduced by FOXO1 knock down. This independence was expected since FOXO1 is known to induce PEPCK. The induction of PEPCK by a known inducer of FOXO1 was confirmed in our HepG2 cells since resveratrol induced PEPCK as expected (see figure 7A). Since the flavones also evoked a nuclear translocation of FOXO1 some additional factors must have altered the effects on the expression of PEPCK resulting in reduced mRNA levels. The inhibitory action was abolished in the knock downs of NRF2, as was already observed for the basal gene expression. The role of NRF2 was similar for PEPCK and G6Pc basal transcription levels and down-regulations by the flavones luteolin and apigenin. We therefore conclude that the interaction of NRF2 with FOXO1 most likely explains the inhibitory effects on the expression of PEPCK and G6Pc. NRF2 is activated by numerous xenobiotics and oxidative stress and was shown to improve hepatic defense mechanisms against metabolic injuries such as high fat diets [30].

A significant down-regulation of gluconeogenic and lipogenic gene expression by apigenin and luteolin in cells from human liver carcinomas (similar results were obtained in parallel experiments with HUH-7 cells, data not shown) make these flavones good candidates for lowering hepatic glucose production (antidiabetic effect) and reducing hepatic steatosis. The consequence of preventing glucose production e.g. by flavone-rich diets during insulin resistance and diabetes offers new possibilities for regulating glucose homeostasis.
